# Azithromycin and doxycycline intravenous and oral administrations before and after prescriber-facing alert for intravenous fluid conservation

**DOI:** 10.1017/ash.2026.10411

**Published:** 2026-06-01

**Authors:** Jennifer Dela-Pena, Jill Wesolowski, Gretchen Sacha, Andrea M. Pallotta

**Affiliations:** Pharmacy, Cleveland Clinic Foundation: Cleveland Clinic, Cleveland, USA

## Abstract

**Objective:**

Intravenous (IV) to oral (PO) antimicrobial stewardship initiatives can be implemented by various healthcare providers in conjunction with clinical decision support tools. Our study evaluated the impact of a prescriber-facing IV to PO alert on azithromycin and doxycycline prescribing, acquisition cost, and waste generated.

**Methods:**

Our institution implemented an IV to PO alert at order entry for IV azithromycin and IV doxycycline with alternative dosage forms included. All emergency department and inpatient IV and PO azithromycin and doxycycline administrations were included during May 12, 2024 – September 28, 2024 (pre-group) and October 13, 2024 – March 1, 2025 (postgroup). The primary outcome was the change in PO azithromycin and doxycycline administrations over time. Secondary outcomes included cost savings and waste generated.

**Results:**

A total of 25,529 azithromycin (56% IV, 43% PO) and 40,584 doxycycline (27% IV, 73% PO) administrations were included in the pre-group. The postgroup included 32,505 azithromycin (22% IV, 78% PO) and 44,601 doxycycline (12% IV, 88% PO) administrations. An increase of almost 38.9% (95% CI 34.9 to 43.0%) in the proportion of PO azithromycin and 17% (95% CI 14.4 to 19.6%) in the proportion of PO doxycycline was observed immediately after alert implementation. Azithromycin and doxycycline total waste decreased by 270.3 and 230.6 kg, respectively. Monthly savings of USD 28,739.48 were calculated for azithromycin and USD 46,637.87 for doxycycline.

**Conclusion:**

A prescriber-facing IV to PO alert led to increase PO administrations of azithromycin and doxycycline with reduction in cost and waste generated.

## Introduction

Drug shortages have clinical and economic consequences such as delayed care, receipt of suboptimal treatment, potential for medication errors, reduced healthcare productivity, and increased cost.^1–3^ Hospitals implement mitigation strategies to avoid or minimize disruption in care.^3^ Due to the severity of the 2024 large-volume sterile fluid shortage, one of the conservation strategies our institution prioritized was the intravenous (IV) to oral (PO) conversion of antibiotics.

Although IV therapy historically has been the preferred route, recent studies demonstrate that PO antibiotics are safe and effective for patients who are clinically stable without concerns for malabsorption.^4–6^ Initiation or transition to PO antibiotics is associated with shorter hospital length of stay, reduced catheter-related complications, and decreased cost.^4–6^ The CDC Core Elements highlights IV to PO antibiotic conversion as a stewardship initiative.^7^

Implementation of IV to PO services involve prescribers, pharmacists, and nurses. Clinical decision support tools integrated into the electronic health record help identify patients for intervention review. Our study evaluates changes in IV azithromycin and IV doxycycline administrations and financial impact with acquisition cost and waste generated following implementation of a prescriber-facing IV to PO alert encouraging IV fluid conservation.

## Methods

### Description of the alert

**Figure 1.**
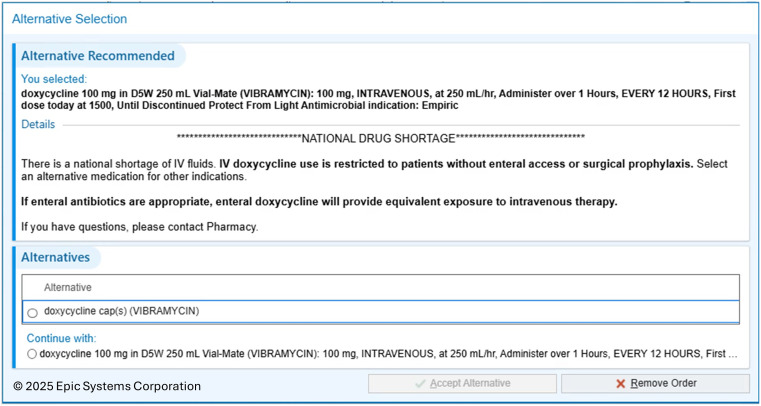
IV Doxycycline alert.

**Figure 2.**
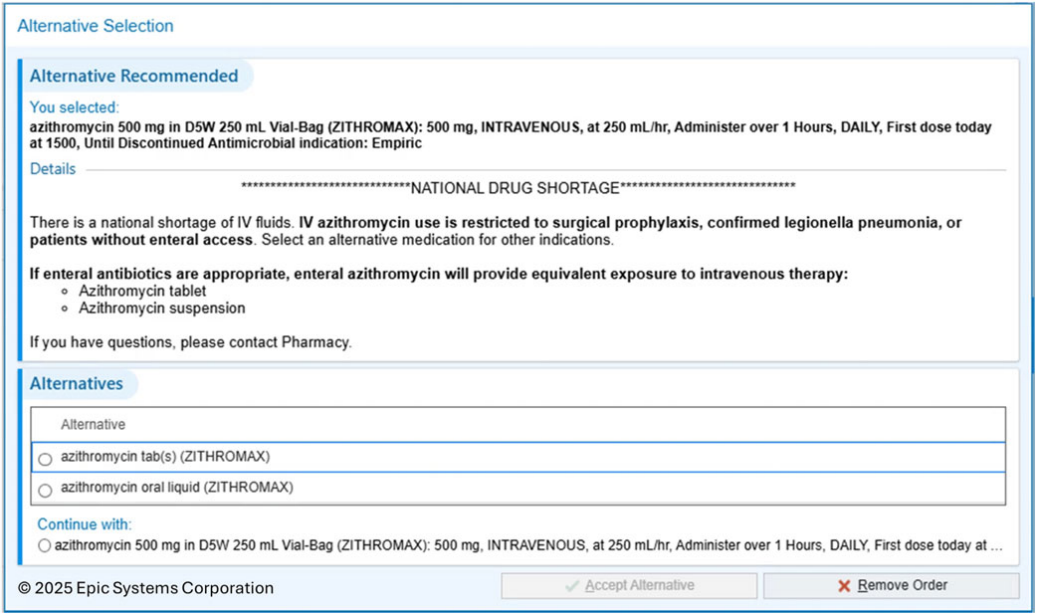
IV Azithromycin alert.

On October 10, 2024, our health system, including 20 hospitals and 7 free-standing emergency departments, implemented an IV to PO alert at time of order signature for every IV azithromycin and IV doxycycline with details on the current large-volume sterile fluid shortage and alternative dosage forms (Figures 1 and 2). The prescriber could select an alternative dosage form or continue with the IV order based on the patient’s clinical characteristics. Our institution has a pharmacist-directed IV to PO conversion service including azithromycin and doxycycline with strict criteria for change. One criterion requires at least 24-hours of IV medication before pharmacist switch to oral. Alternatively, the alert described above triggers at order entry.

### Study design

Adult and pediatric inpatient and emergency department IV and PO azithromycin and doxycycline administrations were included in the pregroup (May 12, 2024 – September 28, 2024) or postgroup (October 13, 2024 – March 1, 2025). These dates were selected to have equal number of weeks pre and postalert implementation. Oral administrations included by mouth and enteral tube routes. Routes of administration other than PO and IV were excluded. IV and PO administrations for each agent were grouped into two-week intervals.

### Statistical analysis

To evaluate the primary objective of impact of an IV to PO alert for azithromycin and doxycycline on oral antibiotic ordering over time, quasi-experimental analyses utilizing interrupted time series analyses (also known as segmented regression) were conducted.^8^ This method uses aggregate data collected over equally spaced time intervals to compare differences in slopes between regression segments both before and after alert implementation and evaluates change in level of outcome (proportion of patients who had either azithromycin or doxycycline ordered orally) immediately before alert was implemented compared with immediately after alert was implemented (regression segment) as well as change in slope before and after alert was implemented. This analysis is recommended for evaluation of longitudinal data sets when comparing pre/postupdate outcomes and accounts for preupdate slope trends.^9–11^ To conduct the interrupted time series analysis model, an ordinary least-squares model with Newey-West standard error was utilized. Autocorrelation was assessed with the Cumby-Huizinga general test and nonstationarity was assessed with modified Dickey-Fuller t-test.

To evaluate secondary end point of total cost savings of azithromycin and doxycycline, we subtracted postcost data from precost data. Cost was calculated using average wholesale price (AWP) in Lexidrug^TM^ at time of evaluation for each medication administered, including IV fluids.^12–15^ If a range was provided, median AWP was used.

The amount of waste generated from IV and PO formulations, empty IV bags, vials, and attachments for IV formulations and empty blister packs or standard oral formulations (azithromycin 500 mg and 250 mg and doxycycline 100 mg) were weighed. Total weight of standard dosage forms in postgroup was subtracted from total weight in pregroup to determine the difference. All analyses were conducted using STATA® (version 13.1, College Station, Texas).^8^

## Results

In the pregroup, 25,529 azithromycin administrations were included with 14,457 (56%) IV and 11,072 (43%) oral. In the postgroup, 32,505 azithromycin administrations were included with 7,258 (22%) IV and 25,247 (78%) oral.

In the pregroup, 40,584 doxycycline administrations were included with 10,763 (27%) IV and 29,821 (73%) oral. In the postgroup, 44,601 doxycycline administrations were included with 5,551 (12%) IV and 39,050 (88%) oral.

**Figure 3.**
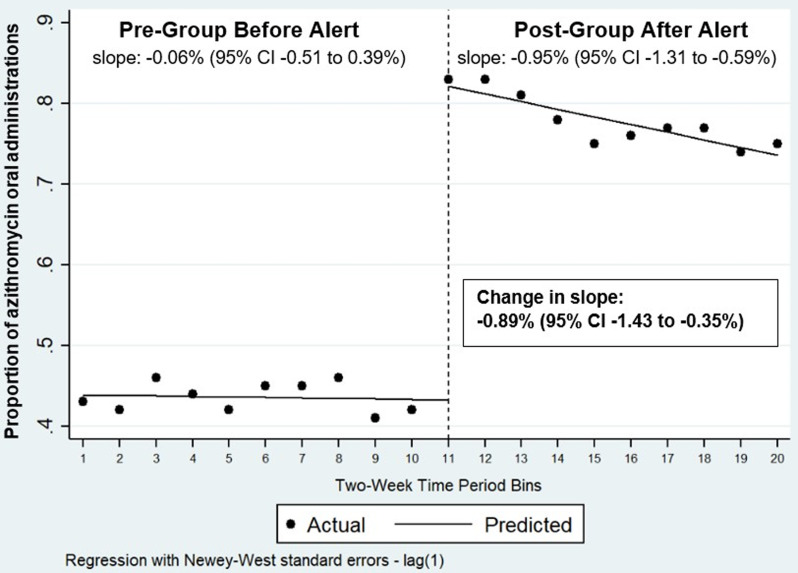
Proportion of azithromycin oral administration before and after implementation of the alert. **Interpretation**: Interrupted time series analysis of the proportion of azithromycin orders that were ordered for oral administration every 2 weeks beginning 5/12/2024 through 3/1/2025. The dashed vertical line represents the point in time in which the IV to PO BPA was implemented (interruption point = 10/13/2024). Data points represent the proportion of azithromycin orders that were ordered for oral administration during the 2-week period; data points on the left side of the interruption point indicate the proportion of azithromycin orders that were ordered for oral administration before the BPA was implemented, and data points on the right indicate the proportion of azithromycin orders that were ordered for oral administration after the BPA was implemented. Lines represent the regression lines for the proportion of orders for oral administration during the study period before and after BPA implementation.

**Figure 4.**
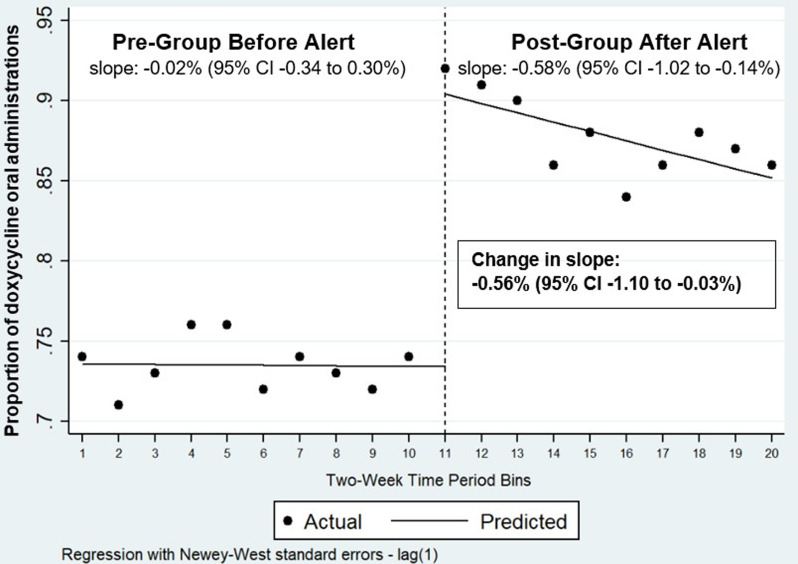
Proportion of doxycycline oral administration before and after implementation of the alert. **Interpretation**: Interrupted time series analysis of the proportion of doxycycline orders that were ordered for oral administration every 2 weeks beginning 5/12/2024 through 3/1/2025. The dashed vertical line represents the point in time in which the IV to PO BPA was implemented (interruption point = 10/13/2024). Data points represent the proportion of doxycycline orders that were ordered for oral administration during the 2-week period. Lines represent the regression lines for the proportion of orders for oral administration during the study period before and after BPA implementation.

Interrupted time series analysis of proportion of orders that were ordered for oral administration are shown in Figure 3 for azithromycin and Figure 4 for doxycycline.

For azithromycin, results showed a premodifier slope of −0.06% (95% CI −0.51 to 0.39%) every 2 weeks and postmodifier slope of −0.95% (95% CI −1.31 to −0.59%) every 2 weeks. There was a significant change in slope detected after alert implementation (change in slope: −0.89% [95% CI −1.43 to −0.35%]). After alert was implemented, there was a significant level change in proportion of azithromycin orders for oral administration: 38.9% (95% CI 34.9 to 43.0%).

For doxycycline, results showed a premodifier slope of −0.02% (95% CI −0.34 to 0.30%) every 2 weeks and a postmodifier slope of −0.58% (95% CI −1.02 to −0.14%) every 2 weeks. There was a significant change in slope detected after alert was implemented (change in slope: −0.56% [95% CI −1.10 to −0.03%]). After alert was implemented, there was a significant level change in the proportion of doxycycline orders for oral administration: 17.2% (95% CI 14.4 to 19.6%).

Total cost of azithromycin decreased from USD 673,663.14 to USD 529,965.75 with monthly savings of USD 28,739.48. The same trend was observed for doxycycline with reduction in total cost from USD 741,947.68 to USD 508,758.21 with monthly savings of USD 46,637.87.

Total amount of waste generated from azithromycin decreased from 550.4 kg in the pregroup to 280.1 kg in the postgroup for a total waste reduction of 270.3 kg. The total amount of waste generated from doxycycline decreased from 493.4 kg in pregroup to 262.8 kg in postgroup for total waste reduction of 230.6 kg.

## Discussion

We describe increased proportion of oral azithromycin and doxycycline administered before and after implementation of prescriber-facing IV to PO alert, leading to cost- and waste-savings.

Beique et al describe concerns about IV to PO conversion, including not understanding the benefit of conversion, misconceptions that PO dosage form yields lower concentration, patients have better clinical outcomes with IV therapy, intensive care unit admission, or presence of a feeding tube.^16^ We attempted to mitigate concerns with the verbiage in our alert and by offering oral capsule or tablet as well as oral solution dosage form selection options, when available.

Various methods of IV to PO conversion have been implemented with earlier interventions involving pharmacists contacting providers to discuss recommendations, which can be time-consuming and require dedicated personnel.^17,18^ One example by Akhloufi et al describe an IV to PO switch alert report generated at 84 hours after prescribing and sent to pharmacists for assessment. The pharmacist contacted prescribers to change from IV to PO or stop antibiotic, if appropriate. Only about 10% of alerts led to a change in therapy.^19^ In contrast, Quintens et al describe implementation of more advanced clinical rules for screening patients for IV to PO switches. Clinical pharmacists reviewed patients, and communicated recommendations via electronic health record notes. A significant and sustained effect was demonstrated with a 79% reduction in IV prescriptions.^20^

The introduction of pharmacy collaborative practice agreement has streamlined the IV to PO process and decreased IV utilization and cost.^21–23^ However, these protocols depend on restriction criteria set forth by hospitals including patient characteristics, clinical parameters, and required documentation of changes. In contrast to our study where patients may receive initial PO antibiotics, most protocols typically require receipt of at least one IV dose or improvement in infectious signs or symptoms. Jaggar et al describe an increase in IV to PO conversion and cost reduction when a preexisting protocol was modified to be less restrictive to increase eligibility for the intervention.^24^

Prescriber-facing alerts are a strategy for IV to PO initiatives with implementation of computerized provide order entry systems.^25,26^ However, the rate of conversion is lower in comparison to pharmacist-managed protocols. Concern about alert fatigue and bypassing alerts based on location and acuity of patients have been previously described. Galanter et al observed a low overall compliance rate of 19% with only 15% in the intensive care units in comparison to 21% in the medical-surgical wards.^26^ In contrast, although our study did not evaluate compliance rate, we did see a significant reduction in IV utilization postimplementation of the alert. This may be due to the nature of the alert with the IV fluid shortage and the option for prescribers to change to oral therapy from the alert without additional steps. Additionally, IV azithromycin is included on sepsis order-sets and likely selected more often for initial therapy, leading to potential greater impact when the prescriber-facing alert was implemented compared to doxycycline.

Our study is not without limitations. We described our health-system experience, which may not translate to other practice areas. We could not assess the number of alerts not resulting in IV to PO change. The adoption of the alert during national shortage may have been led to higher acceptance earlier during the shortage. We did not assess durability of PO switch. Although previously described in the literature, we could not assess clinical outcomes of the IV to PO switch.^4–6^

In conclusion, a prescriber-facing IV to PO alert resulted in decrease utilization of IV formulation, acquisition cost, and waste generated. With the observed benefits of the IV to PO alert, our institution is working on adjusting the alert language following the resolution of the large-volume IV fluid shortage. A work group has also been tasked in evaluating other medications that may qualify for the prescriber-facing alert.
